# Impact of antenatal corticosteroid exposure on glycemic control in preterm infants assessed by continuous glucose monitoring: preliminary results from a single center study

**DOI:** 10.3389/fendo.2026.1847988

**Published:** 2026-10-02

**Authors:** Irene Bonato, Andrea Calandrino, Samuele Caruggi, Marcella Battaglini, Francesco Vinci, Gaia Cipresso, Carolina Montobbio, Chiara Andreato, Giorgia Brigati, Luca Antonio Ramenghi

**Affiliations:** 1 Neonatal Intensive Care Unit, Department of Maternal and Neonatal Health, IRCCS Istituto Giannina Gaslini, Genoa, Italy; 2 Department of Neuroscience, Rehabilitation, Ophthalmology, Genetics, Mother and Child Health, School of Medical and Pharmaceuticals, University of Genoa, Genoa, Italy

**Keywords:** antenatal corticosteroids (ACS), continuous glucose monitoring (CGM), glucose variability, hyperglycemia, hypoglycemia, preterm infants

## Abstract

**Background:**

Antenatal corticosteroids (ACS) are routinely administered to women at risk of preterm delivery to improve neonatal respiratory outcomes. However, ACS exposure has been associated with neonatal hypoglycemia, and its impact on overall glycemic control and variability—particularly at earlier gestational ages—remains incompletely defined.

**Objectives:**

To evaluate glycemic control using continuous glucose monitoring (CGM) in preterm infants exposed to ACS and to assess the influence of timing of administration and gestational age on glycemic patterns.

**Methods:**

This prospective single-center cohort study included preterm infants born at <35 weeks of gestation and admitted to a level III neonatal intensive care unit between February 2023 and August 2024. Infants were classified according to ACS exposure as complete course, partial course, or no exposure. CGM was performed during the first 48 hours of life. Hypoglycemic (<45 mg/dL) and hyperglycemic (>180 mg/dL) events, as well as glycemic variability assessed by the mean amplitude of glycemic excursions (MAGE), were analyzed. Subgroup analyses were conducted based on the interval between ACS administration and delivery (≤7 days vs >7 days) and gestational age (<34 vs ≥34 weeks). Statistical significance was adjusted using the Benjamini–Hochberg procedure.

**Results:**

Sixty-four infants were included (41 complete ACS, 15 partial ACS, 8 no ACS). Baseline characteristics were comparable among groups. During the first day of life, hypoglycemic events occurred exclusively in infants exposed to a complete course of ACS (p = 0.005), predominantly in those exposed within seven days prior to delivery. This difference was not observed on the second day. Hyperglycemic events were significantly more frequent in infants born before 34 weeks of gestation (p = 0.005). Glycemic variability, assessed by MAGE, did not differ significantly according to ACS exposure, timing, or gestational age.

**Conclusions:**

Exposure to a complete and recent course of ACS is associated with an increased risk of early neonatal hypoglycemia in preterm infants, particularly within the first 24 hours of life. CGM enables detailed assessment of glycemic instability and may support improved metabolic monitoring in this high-risk population.

## 1 Introduction

The administration of antenatal corticosteroids (ACS) to women at risk of preterm birth has long been recognized as one of the most effective interventions to reduce neonatal mortality and morbidity associated with respiratory distress syndrome. While concerns have been raised regarding potential short- and long-term adverse effects in newborns exposed to repeated courses of steroids, a single course of ACS is currently considered safe and remains standard clinical practice according to international guidelines (1).

Among reported adverse effects, neonatal hypoglycemia has been described following ACS exposure, particularly in late preterm infants (2–4). The Antenatal Late Preterm Steroids (ALPS) trial by Gyamfi Bannerman et al. (2) was the first large randomized study to demonstrate both the respiratory benefits of ACS administration in late preterm infants and an increased risk of neonatal hypoglycemia in exposed newborns compared with unexposed controls. Subsequent studies have confirmed this association, reporting a higher incidence of hypoglycemia not only in late preterm infants but also in those born at earlier gestational ages (5, 6).

Although neonatal hypoglycemia is generally amenable to prompt and effective treatment, the impact of mild, transient, and asymptomatic hypoglycemia on the developing brain remains uncertain. In contrast, prolonged or recurrent neonatal hypoglycemia has been associated with adverse neurological outcomes, including developmental delay, seizures, visual processing deficits, and cognitive impairment (7). Neonatal hypoglycemia has also been associated with a dose-dependent increased risk of impaired executive function and visual–motor integration in neurodevelopmental assessments performed at 4.5 years of age (8). A recent meta-analysis evaluating neurodevelopmental outcomes following neonatal hypoglycemia reported an increased risk of visual–motor impairment and executive dysfunction in early childhood, as well as literacy and numeracy difficulties later in life (9).

In addition to hypoglycemia, increasing attention has been directed toward neonatal hyperglycemia and glycemic variability (GV), which encompasses both upward and downward acute glucose fluctuations. Elevated glycemic variability and hyperglycemic episodes have been associated with increased oxidative stress, leading to cellular damage and apoptosis (10). This is particularly relevant in preterm infants, in whom oxidative stress plays a key role in the pathogenesis of several major neonatal morbidities, including periventricular leukomalacia, bronchopulmonary dysplasia, and retinopathy of prematurity (11).

For these reasons, strict glucose monitoring is essential to prevent and promptly manage glucose instability in preterm newborns. Current standard care relies on intermittent capillary blood glucose measurements, typically performed at least eight times during the first 24 hours of life (12).

More recently, continuous glucose monitoring (CGM) systems—widely used in adults and children with diabetes—have been introduced into neonatal care. CGM measures interstitial glucose concentrations via a subcutaneous sensor, providing continuous real-time glucose profiles.

Several studies have demonstrated that CGM is feasible, reliable across a wide range of glucose values, and potentially less painful than repeated capillary sampling in preterm infants (13, 14). Importantly, continuous monitoring may allow the detection of silent or asymptomatic hypoglycemic episodes that might otherwise remain undiagnosed, thereby potentially reducing the risk of neurodevelopmental injury (15).

However, despite these advantages, there are currently no formal recommendations for CGM use in neonatal intensive care units, and its clinical interpretation remains challenging due to the dynamic nature of glucose regulation in newborns (16). In this context, observational studies using CGM may contribute to defining clinically relevant glycemic patterns and support future neonatal applications.

The primary aim of the present study was to evaluate glycemic control using continuous glucose monitoring in preterm infants exposed to antenatal corticosteroids for lung maturation. Secondary objectives were to assess whether glycemic trends differed according to the interval between ACS administration and delivery, and according to gestational age, using a cutoff of 34 weeks, as most previous studies have focused predominantly on late preterm infants.

## 2 Materials and methods

### 2.1 Study design and population

This prospective single-center cohort study was conducted in the level III Neonatal Intensive Care Unit (NICU) of the Giannina Gaslini Institute, Genoa, Italy.

Preterm infants with a gestational age <35 weeks and a birth weight >1000 g, admitted to the NICU between February 2023 and August 2024, were eligible for inclusion. Recruitment included both inborn infants delivered at the Obstetrics Department of the Giannina Gaslini Institute and outborn infants transferred to the unit for specialized neonatal care, within the first 6 hours of life.

Exposure to antenatal corticosteroids (ACS) administered for fetal lung maturation was assessed for each pregnancy. Exposure variables included completion of the steroid course (complete vs partial) and the interval between ACS administration and delivery.

In accordance with local and national guidelines, antenatal corticosteroid prophylaxis is routinely offered to women between 24 and 34 weeks of gestation who are at risk of preterm delivery within seven days (17). The standard regimen consists of two intramuscular doses of betamethasone (12 mg each) administered 24 hours apart (1).

### 2.2 Exclusion criteria

Infants were excluded if they had a prenatal or postnatal diagnosis of major congenital anomalies, metabolic, genetic, or endocrine disorders, neonatal hypoxic–ischemic encephalopathy, or if maternal corticosteroid therapy had been administered for indications other than fetal lung maturation.

Study groups Enrolled infants were categorized into three groups based on antenatal corticosteroid exposure:

- Complete ACS course: two doses of intramuscular betamethasone administered at any time during pregnancy.
- Partial ACS course: one dose of 12 mg betamethasone.
- No ACS exposure: mothers did not receive antenatal corticosteroids due to lack of indication or insufficient time before emergency delivery.
- No ACS exposure: mothers did not receive antenatal corticosteroids due to lack of indication.
- Within the complete ACS group, infants were further stratified according to the interval between steroid administration and delivery:
  O exposure ≤7 days before birth.
  O exposure >7 days before birth.
- Additionally, infants in the complete ACS group were stratified by gestational age at birth:
  O exposure >7 days before birth.
  O <34 weeks.
  O ≥34 weeks.

### 2.3 Data collection and clinical outcomes

Demographic and perinatal data collected included gestational age at birth, birth weight, birth weight centile, sex, plurality, mode of delivery, pregnancy history, and delivery characteristics.

Clinical outcomes evaluated during hospitalization included respiratory support requirements and major neonatal complications, such as sepsis, intraventricular hemorrhage (IVH), and necrotizing enterocolitis (NEC).

### 2.4 Continues glycemic monitoring   

All enrolled infants underwent continuous glucose monitoring (CGM), initiated within the first 6 hours of life and continued for at least 48 hours. On admission, parenteral nutrition was initiated with a glucose infusion rate (GIR) of 4–5 mg/kg/min, in accordance with ESPGHAN guidelines (18).

Real-time CGM was performed using the Medtronic MiniMed Guardian Connect system (MMT-7820WE, Medtronic, Northridge, CA, USA) with Enlite sensors (Medtronic of Canada Ltd., Brampton, Ontario, Canada). Sensors were inserted as early as possible after enrollment.

Following skin disinfection, the sensor was placed in the lateral thigh and secured with a transparent adhesive dressing. The device is minimally invasive, flexible, and disposable, measuring approximately 1 mm in width and 10 mm in length, and is inserted using a hollow needle. To reduce procedural discomfort, 0.5 mL of 10% glucose solution was administered orally prior to insertion in non-sedated infants.

Interstitial glucose levels were recorded every 5 minutes (up to 288 measurements per day). Data were collected for at least 48 hours and downloaded using dedicated software upon sensor removal. Calibration was performed using capillary blood glucose measurements obtained with a FreeStyle Optium Neo H glucometer (Abbott Healthcare, MA, USA), at least three times daily according to the manufacturer’s recommendations.

Alarm thresholds were set at <45 mg/dL for hypoglycemia and >180 mg/dL for hyperglycemia. All clinically relevant CGM alarms were confirmed by capillary blood glucose measurements before therapeutic interventions whenever feasible. In all cases, dysglycemia was managed according to our internal protocol by adjusting the GIR in increments or decrements of 0.5 mg/kg/min. In cases of severe hypoglycemia (<25 mg/dL), a bolus of 10% glucose (2 mL/kg) was administered. Insulin therapy was reserved for persistent hyperglycemia despite reduction of GIR.

In case of sensor dislodgement or malfunction, one replacement was allowed. Monitoring was discontinued in the event of transfer to another unit. Infants were closely observed for local adverse events at the insertion site, including skin irritation, bleeding, or infection. No CGM-related complications were reported, and the device did not interfere with routine clinical care.

### 2.5 Statistical analysis

Descriptive statistics were performed, and data are presented as mean ± standard deviation (SD) or median (range) for continuous variables, and as absolute and relative frequencies for categorical variables.

Categorical variables were compared using the chi-square test or Fisher’s exact test, as appropriate. Continuous variables were analyzed using one-way analysis of variance (ANOVA); when the assumption of homogeneity of variances was violated, Welch’s ANOVA was applied.

For the analysis of continuous glucose monitoring (CGM) data, the number and proportion of hypoglycemic (<45 mg/dL) and hyperglycemic (>180 mg/dL) events were calculated for each group during the first and second days of life. Given that CGM provides repeated measurements within the same individual, glycemic events were analyzed as aggregated counts; therefore, these analyses should be interpreted as exploratory, as within-subject correlation was not explicitly accounted for.

Glycemic variability was assessed using EasyGV software (available for academic use at www.phc.ox.ac.uk/research/technology-outputs/easygv), which calculates several indices of glycemic variability from CGM data. In this study, standard deviation (SD) and mean amplitude of glycemic excursions (MAGE) were reported.

To account for the increased risk of false-positive findings due to multiple comparisons, the Benjamini–Hochberg procedure was applied to control the false discovery rate (FDR). The correction was applied separately within each family of comparisons (corresponding to each table), and adjusted p-values are reported where appropriate. Baseline comparisons were performed for descriptive purposes and should not be interpreted as formal hypothesis testing.

Statistical analyses were conducted using SPSS for Windows, version 20 (IBM Corp., Armonk, NY, USA).

## 3 Results

A total of 64 preterm infants met the eligibility criteria and were included in the analysis. Of these, 41 infants were exposed to a complete course of antenatal corticosteroids (ACS), 15 to a partial course, and 8 were not exposed to ACS.

Baseline demographic and perinatal characteristics of the three groups are summarized in **Table 1**. Overall, the groups were comparable with respect to most baseline variables. No statistically significant differences were observed among groups in gestational age, birth weight, birth weight centile, sex, Apgar scores at 1 and 5 minutes, intrauterine growth restriction (IUGR), mode of delivery, or inborn status after adjustment for multiple comparisons using the Benjamini–Hochberg procedure.

**Table 1:** Clinical characteristics of the study patients.

Baseline characteristics	ACS complete course N= 41	ACS partial course N=15	ACS not administered N=8	P value
Gestational age, weeks, (Mean ± SD)	32.46 ± 1.62	31.45 ± 2.26	33.94 ± 0.93	0,17
Birth weight, (Mean ± SD)	1,564.76 ± 394.94	1,678.33 ± 538.74	1,707.50 ± 609.54	0,5
Centile, (Mean ± SD)	39.15 ± 27.92	50.33 ± 29.81	46.38 ± 25.01	0,3
TWIN (Yes, %)	20 (48.8)	2 (13.3)	0 (0.0)	**0,04**
Sex (M, %)	20 (48.8)	7 (46.7)	4 (50.0)	0,9
APGAR 1, (Mean ± SD)	6.70 ± 2.07	5.87 ± 2.10	6.13 ± 1.64	0,39
APGAR 5, (Mean ± SD)	8.28 ± 1.28	8.07 ± 0.88	7.88 ± 1.13	0,65
IUGR (Yes, %)	11 (26.8)	2 (13.3)	0 (0.0)	0,26

Statistical significance is expressed in bold (p<0.05).

A significantly higher proportion of twin pregnancies was observed in the complete ACS group compared with the partial and no-ACS groups (p = 0.04). Placental abruption was more frequently observed among infants not exposed to ACS (adjusted p = 0.04) (**Table 2**). This finding should be interpreted cautiously given the small size of the no-ACS group. It likely reflects the acute and unpredictable nature of deliveries in which antenatal corticosteroids could not be administered, rather than a direct association between placental abruption and lack of ACS exposure.

**Table 2:** Mode of delivery.

Delivery mode (planned cesarean section) (n, %)	10 (24.4)	1 (6.7)	0 (0.0)	0,32
Delivery mode (urgent cesarean section) (n,%)	22 (53.7)	10 (66.7)	7 (87.5)	0,32
Delivery mode (spontaneous) (n,%)	9 (22.0)	4 (26.7)	1 (12.5)	0,32
Placental abruption (n, %)	4 (9,8)	1 (6,7)	4 (50,0)	**0,04**
inborn (n, %)	15 (36.6)	10 (66.7)	4 (50.0)	0,26

Statistical significance is expressed in bold (p<0.05).

Continuous variables are presented as mean ± SD and categorical variables as n (%). P-values were adjusted for multiple comparisons using the Benjamini–Hochberg procedure within this table. Baseline comparisons are provided for descriptive purposes and should not be interpreted as formal hypothesis testing.

Neonatal complications and respiratory outcomes are summarized in **Table 3**. No statistically significant differences were observed among the three groups in the incidence of sepsis, intraventricular hemorrhage (IVH), or necrotizing enterocolitis (NEC).

**Table 3:** Patients’ complications and respiratory outcome.

Patients’ complications and respiratory outcome	ACS Complete course N= 41	ACS Partial course N = 15	ACS not administered N=8	P value
Sepsis (n, %)	8 (19.5)	4 (26.7)	2 (25.0)	0,8
Intraventricular hemorrhage (n, %)	5 (12.2)	2 (13.3)	0 (0.0)	0,6
Necrotizing enterocolitis (n, %)	0 (0.0)	0 (0.0)	0 (0.0)	
Endotracheal intubation (n, %)	16 (39.0)	7 (46.7)	7 (87.5)	0,11
Day of life to reach spontaneous breathing (Mean ± SD)	15.90 ± 17.96	16.57 ± 20.62	25.00 ± 32.68	0,6

Infants not exposed to antenatal corticosteroids showed a numerically higher rate of endotracheal intubation compared with those exposed to a complete or partial course (87.5% vs 39.0% and 46.7%, respectively); however, this difference did not reach statistical significance after adjustment for multiple comparisons (p = 0.11).

Similarly, the mean number of days required to achieve spontaneous breathing did not differ significantly among the three groups.

Continuous variables are presented as mean ± SD and categorical variables as n (%). P-values were adjusted for multiple comparisons using the Benjamini–Hochberg procedure within this table.

The number of glucose detections and the frequency of hypoglycemic and hyperglycemic events during the first and second days of monitoring are reported in **Table 4**.

**Table 4:** Number of glucose measurements recorded during day 1 and day 2.

Hypoglycemic and hyperglycemic episodes	ACS complete course	ACS partial course	ACS not administrated	P value
number of events detected: 1st day	10464	3443	1627	
< 45 mg/dl (%)	47 (0.4)	0 (0.0)	0 (0.0)	**0.005**
> 180 mg/dl (%)	130 (1.2)	73(2,1)	163(10)	**0.005**
number of events detected: 2nd day	10438	3076	1739	
< 45 mg/dl (%)	7 (0.1)	3 (0.1)	0 (0.0)	**0,5**
> 180 mg/dl (%)	263 (2.5)	0 (0.0)	88 (5)	**0.005**

P-values were adjusted for multiple comparisons using the Benjamini–Hochberg procedure within this table.

Statistical significance is expressed in bold (p<0.05).

During the first day of life, hypoglycemic events (<45 mg/dL) were observed exclusively in infants exposed to a complete course of antenatal corticosteroids (0.4%), whereas no episodes were detected in the partial ACS or no-ACS groups. This difference remained statistically significant after adjustment for multiple comparisons using the Benjamini–Hochberg procedure (p = 0.005).

Hyperglycemic episodes (>180 mg/dL) were also significantly different among groups during the first day of life (p = 0.005), with the highest proportion of events observed among infants not exposed to ACS.

On the second day of monitoring, hypoglycemic events were infrequent and did not differ significantly among groups (p = 0.5). In contrast, hyperglycemic episodes remained significantly different among groups during the second day of monitoring (adjusted p = 0.005). The highest proportion of hyperglycemic events was observed in infants not exposed to ACS, although the absolute number of hyperglycemic events was higher in the complete ACS group, this reflected the substantially larger number of CGM measurements in that group. When expressed as proportions, hyperglycemic events were more frequent in the no-ACS group.

Glycemic variability, assessed using the mean amplitude of glycemic excursions (MAGE), is reported in **Table 5**. No statistically significant differences in MAGE values were observed among the three groups during either the first or second day of monitoring.

**Table 5:** MAGE index to calculate glycemic variability in the three groups.

Index of glycemic variability	ACS complete course	ACS partial course	ACS not administered	P value
Day 1	9.451 ± 12.303	12.633 ± 15.846	7.000 ± 10.043	0,6
Day 2	8.307 ± 9.037	6.944 ± 7.626	8.333 ± 10.877	0,8

P-values were adjusted for multiple comparisons using the Benjamini–Hochberg procedure within this table.

### 3.1 Timing of ACS administration and glycemic outcomes

Among infants exposed to a complete course of ACS (n = 41), 27 were exposed within seven days prior to delivery, while 14 were exposed more than seven days before birth.

As shown in **Table 6**, hypoglycemic events during the first day of life occurred exclusively in infants exposed to ACS within seven days before delivery (0.6%), whereas no episodes were observed in those exposed more than seven days before birth. This difference remained statistically significant after adjustment for multiple comparisons (p = 0.005).

**Table 6:** Hypoglycemic and hyperglycemic episodes and MAGE index in relation to the distance between complete ACS administration and delivery.

Hypoglycemic and hyperglycemic episodes and time of ACS administration	ACS ≤ 7 days (N = 27)	ACS > 7 days (N = 14)	P value
**number of detection 1st day**	7527	2937	
**< 45 mg/dl (%)**	47 (0.6%)	0 (0.0%)	**0,005**
**> 180 mg/dl (%)**	83 (1.1%)	47 (1.6%)	0,1
**number of detection 2nd day**	7811	2627	
**< 45 mg/dl (%)**	7 (0.1%)	0 (0.0%)	0,35
**> 180 mg/dl (%)**	263 (3.4%)	0 (0.0%)	**0,005**
MAGE, Index of glycemic variability			
Day 1	10.128 ± 13.443	9.117 ± 11.270	0,8
Day 2	7.029 ± 8.702	11.133 ± 8.871	0,2

The interval was calculated from completion of ACS administration (second dose) to delivery. P-values were adjusted for multiple comparisons using the Benjamini–Hochberg procedure within this table.

Statistical significance is expressed in bold (p<0.05).

Hyperglycemic episodes during the first day of life did not differ significantly between the two groups (p = 0.1). However, during the second day of monitoring, hyperglycemic events were observed only in infants exposed to ACS within seven days prior to delivery (3.4%), reaching statistical significance (p = 0.005).

Glycemic variability, assessed using the MAGE index, did not differ significantly between infants exposed within seven days and those exposed earlier during either the first or second day of life (.

### 3.2 Gestational age stratification

Within the complete ACS group, 33 infants were born before 34 weeks of gestation and 8 at or beyond 34 weeks. As reported in **Table 7**, hyperglycemic events were significantly more frequent in infants born before 34 weeks during both the first and second days of monitoring (p = 0.005 for both), whereas the frequency of hypoglycemic events did not differ significantly between the two gestational age groups (p = 0.58 and p = 0.67 for days 1 and 2, respectively).

**Table 7:** Hypoglycemic and hyperglycemic episodes and MAGE index in relation to gestational age at birth.

Hypoglycemic and hyperglycemic events and gestational age	< 34 weeks of gestation (N = 33)	≥ 34 weeks of gestation (N = 8)	P value
**number of detection 1st day**	7446	3018	
**< 45 mg/dl (%)**	36 (0.5%)	11 (0.4%)	0,58
**> 180 mg/dl (%)**	121 (1.6%)	9 (0.3%)	**0,005**
**number of detection 2nd day**	7780	2658	
**< 45 mg/dl (%)**	6 (0.1%)	1 (0.0%)	0,67
**> 180 mg/dl (%)**	244 (3.1%)	19 (0.7%)	**0,005**
MAGE, index of glycemic variability			
Day 1	11.261 ± 14.262	6.103 ± 6.960	0,13
Day 2	7.591 ± 9.248	9.098 ± 7.776	0,6

P-values were adjusted for multiple comparisons using the Benjamini–Hochberg procedure within this table.

Statistical significance is expressed in bold (p<0.05).

Glycemic variability, assessed using the MAGE index, was numerically higher in infants born before 34 weeks on day 1 but did not reach statistical significance (p = 0.13), and no differences were observed on day 2 (p = 0.6).

## 4 Discussion

The present study demonstrates that exposure to a complete course of antenatal corticosteroids is associated with an increased risk of neonatal hypoglycemia in preterm infants, particularly during the first 24 hours of life. This effect appears to be strongly related to the timing of ACS administration, with hypoglycemic events occurring almost exclusively in infants exposed within seven days prior to delivery.

These findings are consistent with previous evidence from the Antenatal Late Preterm Steroids (ALPS) trial, which first reported an increased incidence of neonatal hypoglycemia following ACS administration in late preterm infants (2). Subsequent studies and meta-analyses have confirmed this association (3, 4, 19). Our data further support and extend these observations by showing that the increased risk of hypoglycemia is not limited to late preterm infants but is also present at earlier gestational ages, as previously suggested by Di Pasquo et al. (6).

However, while Di Pasquo et al. identified gestational age as the principal determinant of hypoglycemia and did not demonstrate a significant influence of ACS timing or completeness (6), our data suggest that both recent exposure and completion of the steroid course may contribute to hypoglycemia risk. This discrepancy may be explained by differences in study populations and by the use of continuous glucose monitoring in our cohort, which allowed the detection of transient and asymptomatic hypoglycemic episodes that might have been missed by intermittent glucose sampling.

With regard to the timing of hypoglycemia onset, previous studies have mainly reported an increased risk within the first 12 hours of life (20). Our findings indicate that hypoglycemic events occurred within the first 24 hours of life; however, the study design does not allow discrimination between early (0–12 h) and later (12–24 h) onset, and therefore the temporal pattern within this period cannot be further specified.

The mechanisms underlying ACS-related neonatal hypoglycemia remain incompletely understood. One proposed mechanism involves maternal hyperglycemia induced by corticosteroid administration, leading to fetal hyperinsulinemia and subsequent postnatal hypoglycemia (5). Another hypothesis suggests suppression of the fetal hypothalamic–pituitary–adrenal axis, resulting in an impaired cortisol-mediated stress response after birth (21). Finally, glucocorticoids may influence the maturation of the fetal sympathoadrenal system and catecholamine synthesis, which play a key role in metabolic adaptation at birth (22).

Although hormonal measurements were not available in our study, the temporal association between recent ACS exposure and hypoglycemic events supports a direct metabolic effect.

In addition to hypoglycemia, our study highlights the occurrence of hyperglycemic events, particularly among infants exposed to ACS within seven days of delivery and among those born before 34 weeks of gestation. Hyperglycemia and increased glycemic variability are of clinical interest because of their association with oxidative stress (10), which plays a critical role in the pathogenesis of major preterm morbidities, including bronchopulmonary dysplasia, periventricular leukomalacia, and retinopathy of prematurity (11).

Glycemic variability was assessed using MAGE, a metric designed to capture major glucose excursions independently of mean glucose levels (23). Although MAGE did not differ significantly according to ACS exposure or timing, infants born before 34 weeks of gestation exhibited higher variability during the first day of life. This finding likely reflects the intrinsic metabolic immaturity of very preterm infants, characterized by reduced insulin-sensitive tissue mass, limited glycogen stores (24), and a greater susceptibility to iatrogenic hyperglycemia related to intravenous glucose administration (25). Importantly, in our clinical protocol all episodes of hypoglycemia and hyperglycemia were managed using the same standardized approach, consisting of an increase or decrease in the glucose infusion rate. This uniform management strategy minimizes potential treatment-related variability in glycemic patterns and allows a more reliable interpretation of the observed differences between exposure groups.

An additional aspect to consider is the balance between the well-established benefits of ACS and their potential metabolic side effects across different gestational ages. ACS administration provides clear pulmonary benefits by accelerating fetal lung maturation and has also been associated with a reduction in some neurological complications of prematurity (1, 26). However, the magnitude of these benefits varies according to gestational age. In infants born at very early gestational ages, the reduction in respiratory distress syndrome and severe complications such as intraventricular hemorrhage clearly outweighs potential metabolic adverse effects. Conversely, in infants born at more advanced gestational ages—around 32 weeks, which represents the median gestational age of infants in our cohort—the baseline risk of severe respiratory distress syndrome and major brain injury is substantially lower, whereas metabolic disturbances such as neonatal hypoglycemia may become relatively more relevant. Similar findings have been reported in late preterm infants, where ACS administration improved respiratory outcomes but increased the incidence of neonatal hypoglycemia in the randomized trial published in the New England Journal of Medicine (2).

The main strength of this study lies in the use of continuous glucose monitoring (CGM), which provides a comprehensive and dynamic assessment of glycemic control. Unlike intermittent blood glucose sampling, CGM enables detection of transient hypoglycemic and hyperglycemic events that might otherwise remain unrecognized, thereby potentially reducing the risk of neurodevelopmental injury (15).

CGM has been shown to be safe and feasible in preterm infants and may reduce the need for repeated heel pricks while allowing earlier clinical intervention (27).

This study has several limitations. The relatively small sample size, particularly in the no-ACS group, precluded the use of multivariable regression models to adjust for potential confounders; therefore, the findings should be interpreted as exploratory and require confirmation in larger prospective studies. However, the small sample size in the no-ACS group reflects real-world clinical practice, as antenatal corticosteroids are routinely administered to women at risk of preterm delivery before 34 weeks. Consequently, unexposed infants are typically those born following acute and unpredictable events, such as placental abruption. Importantly, none of these infants showed signs of perinatal asphyxia that could confound glycemic outcomes. Another limitation is that glucose monitoring was restricted to the first 48 hours of life, precluding assessment of longer-term metabolic effects. In addition, the non-blinded design and the fact that glycemic management was actively modified according to CGM readings may have attenuated the magnitude of observed glycemic differences between groups. Specifically, increases in glucose infusion rate in response to hypoglycemia may have reduced the frequency, duration, or severity of subsequent glycemic excursions, potentially leading to an underestimation of the true metabolic effect of antenatal corticosteroid exposure. Furthermore, detailed data on therapeutic interventions, including longitudinal changes in glucose infusion rate, were not systematically collected and therefore could not be included in the analysis.

## 5 Conclusions

In conclusion, exposure to a complete and recent course of antenatal corticosteroids is associated with an increased risk of early neonatal hypoglycemia in preterm infants, particularly within the first 24 hours of life. Although hypoglycemia represents a relatively minor adverse effect compared with the well-established respiratory benefits of ACS, these findings highlight the importance of strict and prolonged glucose monitoring in this population. Continuous glucose monitoring may represent a valuable tool to improve early detection of dysglycemia and optimize metabolic management in preterm infants exposed to antenatal corticosteroids.

## Data Availability

The raw data supporting the conclusions of this article will be made available by the authors, without undue reservation.
